# The Dominant Folding Route Minimizes Backbone Distortion in SH3

**DOI:** 10.1371/journal.pcbi.1002776

**Published:** 2012-11-15

**Authors:** Heiko Lammert, Jeffrey K. Noel, José N. Onuchic

**Affiliations:** Center for Theoretical Biological Physics and Department of Physics, Rice University, Houston, Texas, United States of America; University of Missouri, United States of America

## Abstract

Energetic frustration in protein folding is minimized by evolution to create a smooth and robust energy landscape. As a result the geometry of the native structure provides key constraints that shape protein folding mechanisms. Chain connectivity in particular has been identified as an essential component for realistic behavior of protein folding models. We study the quantitative balance of energetic and geometrical influences on the folding of SH3 in a structure-based model with minimal energetic frustration. A decomposition of the two-dimensional free energy landscape for the folding reaction into relevant energy and entropy contributions reveals that the entropy of the chain is not responsible for the folding mechanism. Instead the preferred folding route through the transition state arises from a cooperative energetic effect. Off-pathway structures are penalized by excess distortion in local backbone configurations and contact pair distances. This energy cost is a new ingredient in the malleable balance of interactions that controls the choice of routes during protein folding.

## Author Summary

Proteins are involved in all aspects of life. To perform their biological function, each protein chain has to fold into a unique three-dimensional structure. This conformational search can only succeed because all interactions are optimized by evolution to create a smooth funnel-shaped energy landscape with a consistent native bias. With the energetics thus leveled, geometrical constraints come to dominate the folding mechanism. But the same interactions that shape the folding mechanism also shape the functional dynamics of the folded protein. Specific functional requirements can thus introduce frustrated interactions that will show up in turn as anomalies in the folding process. In order to extract this functional information a quantitative understanding of the minimally frustrated folding landscape is required. We have devised a method to quantify the contributions from entropy and energy terms that determine the folding mechanism in a model with a perfectly funneled energy landscape. We find for an SH3 domain that its folding mechanism is determined by the energy cost for distortions of the backbone, a term that is usually disregarded in qualitative discussions of protein folding. In general, by counterbalancing the expected dominance of the configurational entropy, the backbone potential should facilitate functional adjustments of the folding mechanism.

## Introduction

Energy landscape theory explains how the various interactions in a protein are organized in order to achieve efficient and robust folding capability [1]–[3]. Protein energy landscapes possess a smooth funnel shape that avoids trapping in non-native states and that guides the folding process to the native structure. These funneled landscapes arise from interactions that are optimized according to the principle of minimal frustration [2], such that the native state is uniquely stabilized by mutually compatible and favorable interactions, while interactions that would favor non-native competing structures are eliminated. The dominance of native interactions indicates that the folding mechanisms are primarily determined by the geometry of the protein's native structure.

Analytical calculations have confirmed the viability of this scenario and helped to understand its wide implications [4]–[10]. The funneled landscape has been realized in minimalist protein models called *structure-based models* [11]–[14], which define completely unfrustrated interactions based on a known native structure. Simulations with structure-based models reproduce both experimental trends [15], [16] and the observed folding mechanisms of many individual proteins [11], [12], [17]–[20].

Furthermore, functional protein dynamics in the native state explore the same funneled energy landscape that controls folding [21], [22]. Minimally-frustrated models should therefore also describe protein function, especially if the scale of relevant motions is not too small compared to the remaining roughness of the natural landscape. Indeed, structure-based models have been adapted to study signalling in the native state [23], limited rearrangements like the opening of the binding pocket in adenylate kinase [24], and entire molecular machines, like motor proteins [25] and the ribosome [26]. These models show that large-scale conformational changes supported by the native folding landscape are facilitated by local unfolding, or cracking [24].

Known exceptions from a perfect funnel, where native interactions alone cannot explain experimentally observed folding mechanisms, have been identified in proteins with analogous structures that fold by different mechanisms [18], [27], [28]. Such departures from a minimally frustrated mechanism have received special attention because they suggest the presence of conflicting functional demands on the protein sequence. In the proteins Im7 and 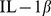, specific functional roles related to ligand binding and receptor signalling have been traced to the frustrated regions that are perturbing their folding mechanisms [18], [29]. Although proteins are susceptible to such functional perturbations of their folding behavior, energetic frustration in general appears to be sufficiently small to let geometrical effects dominate the folding mechanism and functional dynamics. These alternative routes are still consistent with the native geometry, but are suppressed by a few kcal/mol. Subtle changes in perfectly funneled models, like adding side chains [17], can switch the dominant route through the transition state ensemble. But since these models are based on effective interactions, it is quite difficult to specify the particular factors that determine the *dominant* folding mechanism of a minimally frustrated protein.

While individual folding paths on a funneled landscape are highly diverse [30], [31], the observed average folding mechanism of a protein is typically well constrained compared to the vast combinatorial number of possible routes. The order of events cannot be unique at the level of individual contacts because of thermal fluctuations, and the multitude of accessible chain conformations in the unfolded state introduces substantial diversity early in the folding process. But structural preferences have been identified already in molten globule states [32]–[35], and the mechanism becomes more constrained as folding progresses. In the simplest case the protein folds directly through a transition state ensemble that also contains specific features of the native structure. More complex mechanisms may involve specific intermediates. Elements of the native structure that are missing in the transition state finally form to complete the folding process. In a structure-based model the folding mechanism can be specified by the order in which groups of native contacts are formed as the protein folds. In particular, the pattern of formed contacts in the transition state ensemble can be related to experimentally determined 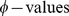 [36].

Folding mechanisms in structure-based models are not directly encoded in the contact potentials, because they assign exactly the same stabilization to each contact. Instead, chain connectivity and excluded volume are the most conspicuous features of a structure-based model that can impose geometrical constraints on a folding protein, and their relevance for the folding mechanism is supported by numerical calculations. Chain connectivity influences the order of contact formation via differences in loop entropy. To minimize the loss of configurational entropy at each stage of folding, contacts should form in order of sequence separation, starting with the contacts that close the shortest loops. Such a mechanism is observed for the very simple case of CI2 [11], [36]. When effective loop lengths are considered, which take into account the effect of previously formed contacts, loop entropy can in principle also lead to more complex mechanisms [5]. Excluded volume is also often considered as an entropic term, because the steep repulsive potential used to enforce it makes conflicting configurations essentially unavailable.

While it is straightforward to understand qualitatively how each of these geometrical factors, as well as specific interactions, can affect protein folding mechanisms, in this study we try to directly compare the influence of geometrical factors and energetic frustration to the folding of an actual protein and determine their contributions in a quantitative way. We focus on the folding of the SH3 domain, a small protein with a simple, but non-trivial, folding mechanism that is successfully reproduced by structure-based models [11], [37], [38]. The transition state ensemble is not obviously dominated by loop entropy like that of CI2, instead structure formation proceeds unevenly (*i.e.* polarized) along the protein chain. The folding mechanism of SH3 though must be determined by the geometry of its fold, because SH3 domains with dissimilar sequences are known experimentally to fold by the same mechanism [38].

The paper is organized as follows: We outline a structure-based model for SH3, and describe the folding process on a two dimensional free energy landscape with coordinates chosen to reflect the folding mechanism. We then introduce a decomposition of the landscape that extracts the entropy and energy contributions that are relevant for the mechanism. This analysis reveals that the mechanism is determined not by entropic effects but by energetic factors. We will demonstrate how this energetic effect arises from distortions of the backbone and imperfections in contact formation, which are only present in an explicit protein model with a direct representation of the chain.

## Methods

### Model

We use a coarse-grained structure-based model that represents each protein residue by a single bead placed at the position of its 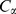 atom [11], [14].
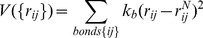
(1.1)

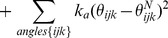
(1.2)

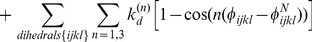
(1.3)

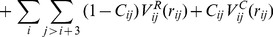
(1.4)All interactions are centered at the known native configuration 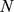. The chain geometry is maintained by harmonic potentials for bonds and angles. Dihedral potentials provide a weak local bias towards native-like torsional angles. Excluded volume is enforced by a repulsive term
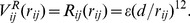
(2)For residue pairs that form a native contact the repulsive term is combined with a Gaussian well placed at the native distance to create an attractive contact potential [13].
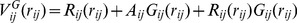
(3)with
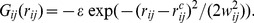
(4)The product term serves to place the minimum of the combined potential at the native distance 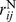. In the potential the parameter 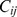 selects the non-bonded interaction for each residue pair. It is one for contact pairs and zero otherwise. The set of native contacts has been determined by the CSU package [39]. Each contact is assigned the same strength 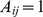. The other interaction constants are 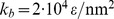, 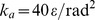, 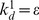 and 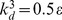, with the reduced unit of energy 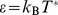, and the size of the beads is 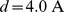.

### System

**Figure 1:**
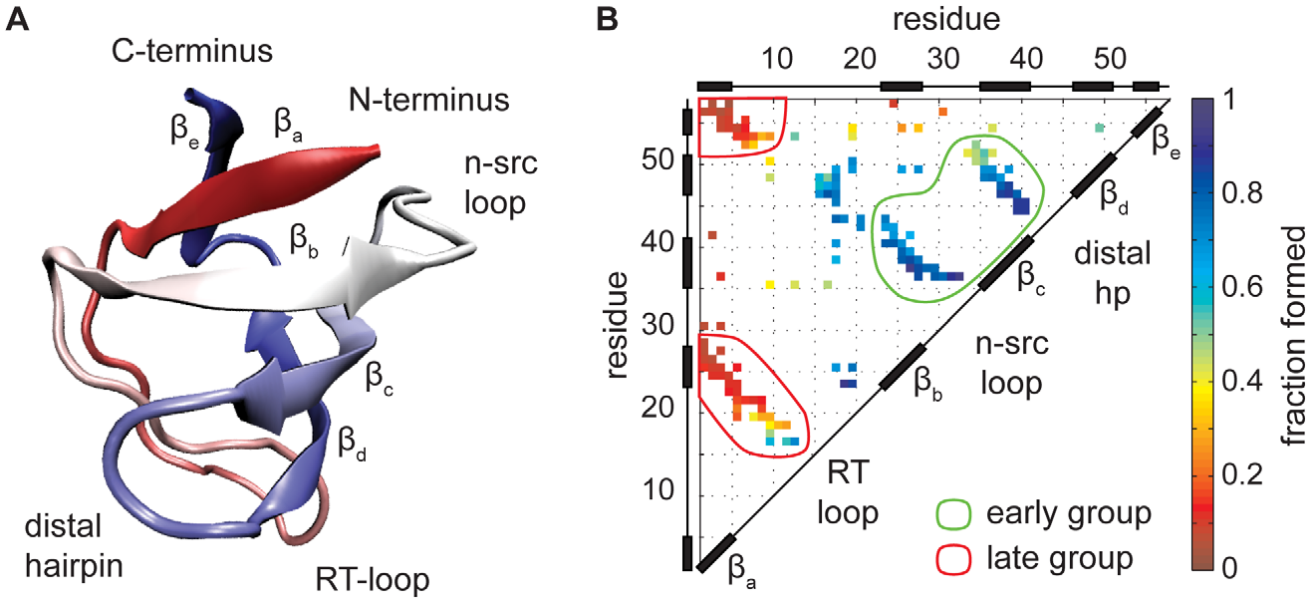
The SH3 domain folds through a polarized transition state. The structure of the domain is shown in **A**. In the contact map **B** all native contacts are shown colored according to their average degree of formation in the transition state, observed during folding simulations using a coarse-grained structure-based model. Native-like local structures are made preferentially in the central beta sheet formed by strands 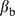, 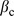 and 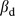, while the terminal strands 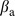 and 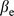 remain mostly unstructured. Specific packing contacts that are relevant for further folding progress are also present, notably between the RT-loop and 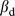. Green and red lines define groups of early- and late-forming hairpin contacts that are chosen to characterise the pattern of formed contacts in individual transition state structures for further analysis.

We apply this simple model to the folding of the SH3 domain of tyrosine kinase c-Src. The folding mechanism of SH3 is experimentally well characterized, and it has often been used as a test case for folding simulations with structure-based models [11], [13]. The structure of c-src SH3 shown in Fig. 1 **A** consists of five beta strands joined in a beta barrel. Our systems comprises 57 residues, numbered 84 to 140 in the PDB structure 1FMK. The model is stabilized by 140 native contacts. The native contacts are organized into four antiparallel beta sheets with additional packing contacts (Fig. 1 **B**). Each contact in the figure is colored according to the probability that it is formed in the transition state ensemble observed with the structure-based model. The pattern agrees qualitatively with the result determined experimentally via mutation studies. In the transition state of the dominant folding route of the wild-type protein, the beta sheet formed by the central strands 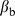, 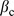 and 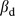 is mostly formed, while the terminal strands 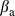 and 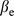 are still mostly unstructured. This situation is commonly described as a polarized transition state, because the progress of structure formation is uneven along the chain. In contrast with expectations based on loop entropy, the folding of the hairpin formed by strands 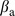, 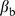 and the intervening RT-loop is delayed compared to contacts with similar sequence separation in the central three-stranded beta sheet.

Because of the unfrustrated potential and the coarse-grained representation of the protein, structure-based models are efficient enough to simulate many folding transitions at equilibrium for a small protein like SH3. The extensive sampling makes it possible to observe rarely visited structures in the transition region.

## Results

To characterize the agreement of observed partially folded structures with the WT-dominant mechanism we focus on the unequal formation of the four groups of beta sheet contacts in the contact map. We assign the contacts of the central three-stranded beta sheet to a group of contacts forming ‘early’, and the contacts of the N- and C-terminal sheets into a second group that is forming ‘late’ in the native mechanism. The quantity 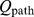 is defined to reflect the difference in folding progress between the two groups in a given configuration,
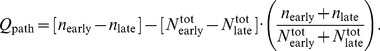
(5)It is basically the difference between the numbers of formed contacts from the early and late groups, 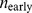 and 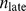. Since 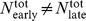, a normalization correction is applied that fixes 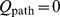 at 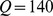. 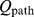 is positive when the pattern of contact formation agrees with the WT-dominant mechanism and contacts from the early group are formed preferentially. Negative values of 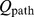 correspond to off-pathway structures with an inverted pattern of contact formation.

### Isolating the free-energy bias in the transition region

**Figure 2:**
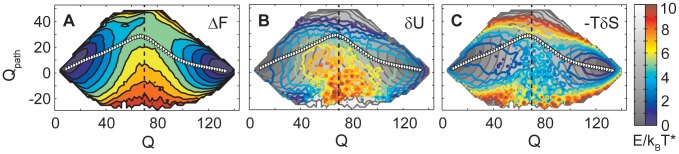
Contributions to the free energy landscape. **A**: Free energy. **B**: Energy contribution 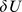. **C**: Entropy contribution 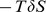. Two dimensional energy landscapes are plotted as a function of the total number of formed contacts 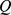 and of 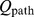, which quantifies the pattern of contact formation. 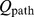 is defined such that positive values correspond to a pattern of contact formation in agreement with the dominant folding route, and negative values mean the inverse preference. White triangles in each plot trace the average folding path that corresponds to the WT-dominant mechanism with its polarized transition state structure. The free energy landscape, shown in panel **A**, has a saddle point between the unfolded and folded basins, located at positive 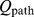. Off-pathway transitions (negative 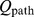) are blocked by a spur of high free energy. The separated energy and entropy contributions are shown in panels **B** and **C**. In gray the full free energy landscape is repeated in the background. The energy contribution 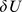, plotted in panel **B**, shows a peak of high energies at low 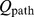 in the transition region, which corresponds to the spur blocking off-pathway transitions in 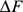. In contrast the entropy contribution 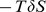, plotted in panel **C**, does not show any feature that would favor a polarized transition state structure.

In Fig. 2 **A** the free energy landscape for the folding of SH3 is plotted as a function of the total number of formed contacts 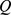 and 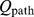. The average reaction path obtained from the simulations, marked by white triangles, passes through a transition state at positive 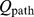. As expected, the WT-dominant folding mechanism corresponds to the route of lowest free energy between the unfolded and native basins. At negative 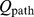 the basins are separated by a region of higher free energy that blocks off-pathway transitions, i.e. those with an inverted mechanism.

Separate plots of the energy and entropy contributions to the free energy landscape should, in principle, reveal directly if the structure in the barrier region that controls the folding mechanism can be traced to either the entropy or the energy term alone. However, in a structure-based protein model both energy and entropy are, by construction, dominated by a strong overall decrease along the main folding coordinate 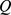. This effect is caused by the progressive formation of native interactions and by the corresponding loss of configurational entropy during folding. The free energy barrier for folding arises from the imperfect cancellation of these terms. Thus, this barrier is much smaller in magnitude than the total change in either energy or entropy. The preference for a particular folding mechanism is determined by a free energy difference that is similarly small compared to the global trends along 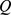. In order to isolate these small features in both contributions that are relevant for the shape of the barrier and for the folding mechanism, the global trend has to be removed. For this purpose we rewrite both the energy and entropy terms 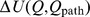 and 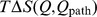 that give rise to the full free energy landscape 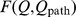. We denote the common component of 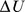 and 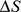 that will cancel out in the subtraction as 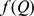, and we define the residual components of 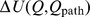 and 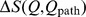 as 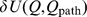 and 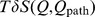.
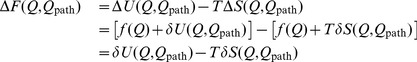
(6)Note that the complete dependence of 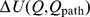 and 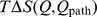 on 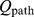 is contained in the residuals 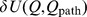 and 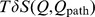. By definition, the common component 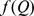 cancels out in the subtraction.

The function 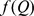 needs to be selected such that it approximates the global trends along 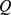 both in 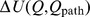 and in 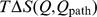. We choose a quadratic function 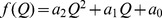 that is determined by a least-squares fit to both 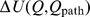 and 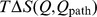 together. This is equivalent to fitting to 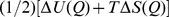. Further details about the fitting are given in the supplementary material.

The residuals 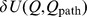 and 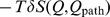 obtained from the described fit are plotted in Fig. 2 **B** & **C** as colored contour lines. The free energy landscape 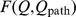 is repeated in gray in the background for reference, along with the average folding pathway. The most favorable values in the transition region of the entropy contribution are located around 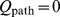. Features that can explain the observed folding mechanism, exist only in the energy contribution. A peak of high energy is located in the transition region at negative 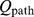.

**Figure 3:**
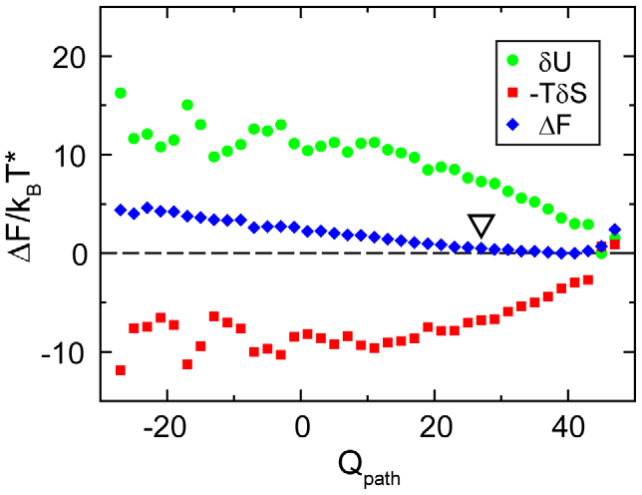
Cross section through the transition region of the free energy landscape. The free energy 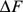 and the contributions 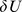 and 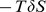 in the transition region at 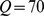 are plotted as a function of the coordinate 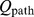, along the dashed line in Fig. 2. Positive values of 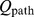 correspond to a native-like pattern of contact formation, negative values signal off-pathway structures with a reversed pattern. The white triangle indicates the position of the average WT reaction path. The bias in the free energy 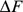 that is favoring WT-dominant transition states is determined by the consistent slope of the energy contribution 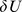. The entropy contribution 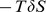 shows a weaker opposite slope towards 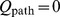 that would penalize the WT-dominant pattern of contact formation.

For further analysis we focus on a cross section through the transition region of the free energy landscape along 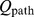 at a constant number of formed contacts, 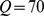, marked in Figs. 2 **A**–**C** by dashed lines. A free energy-bias towards positive transition state structures with positive 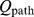 is clearly visible (Fig. 3). The WT-dominant transition state is favored over inverted patterns of contact formation by a total energy difference of up to 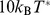. The energy contribution supports this trend, while the entropy contribution shows an opposing trend that penalizes states with positive 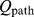. This analysis shows that the energy is the dominant influence on the composition of the transition state, as measured by 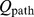.

**Figure 4:**
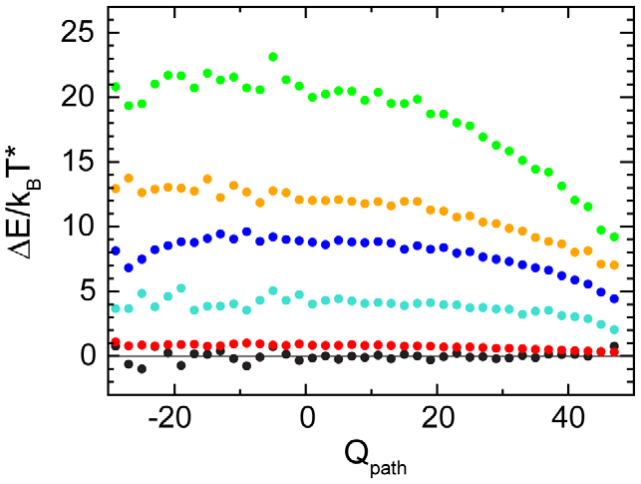
Interactions contributing to the energy bias towards WT-dominant transition states. The potential energy 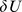 in dependence on 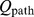, in the transition state region at 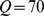, is decomposed into the contributions from each term in the structure-based potential. Data are shifted for visibility. From bottom to top the terms shown are: bonds, repulsion, angles, contacts, dihedrals and the total potential energy. The numerically largest parts of the bias towards a WT-dominant pattern of contact formation in the transition state are provided by dihedral angles and contact potentials, followed by angles. The contibution from repulsive interactions is small but significant. Bond stretching interactions are constant along 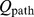.

Individual contributions to the energy along 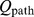 are separated in Fig. 4. All individual energy terms are compatible with the bias displayed by the total energy and strengthen it to different degrees, or are at least neutral. Contacts and dihedral potentials are responsible for the largest part of the effect, each changing by 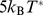 between favored and penalized transition state structures. A smaller contribution also comes from the angle term. Bond stretching energy is the only term that is constant along 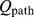 within the errors. Repulsive interactions are small in magnitude, but also show dependence on the pattern of formed contacts.

### Response of the mechanism to modified interactions

The observed differences in contact energy between different states compared in the analysis must arise from differences in the stabilization of formed contacts, because the number of contacts itself is identical in all selected structures. Such differences can arise because the number of formed contacts is counted using a sharp distance cutoff, while the stabilization is determined by a differentiable potential. The stabilization of a contact is already reduced by deviations from the native pair distance that are too small to consider the contact as broken. Average extra deviations of less than 0.2 Å per contact are sufficient to explain the observed destabilization of the contact potential in off-pathway transition states by 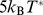 distributed over 70 formed contacts. The increased dihedral and also angular energies found in off-pathway structures correspond in the same way to small additional distortions in their local backbone configurations compared to the WT-dominant transition state.

Backbone configurations and contact distances are mutually dependent, and distortions of the backbone translate necessarily into distortions of contact distances and vice versa. But this geometrical relationship between distortions of the backbone and of the contacts cannot explain either one of them. The unfrustrated construction of the native-centered potential means that both terms could be relaxed simultaneously, if no additional force opposes this relaxation. The increased repulsive energies observed in the off-pathway structures suggest that more favorable configurations are not reached because of excluded volume. Numerically, the contribution from repulsive energies to the observed penalty for off-pathway structures is weak, but as the only unspecific and potentially non-native term in the system, the repulsive interactions have a unique capacity to maintain distortions and thus trigger penalties from other interactions.

**Figure 5:**
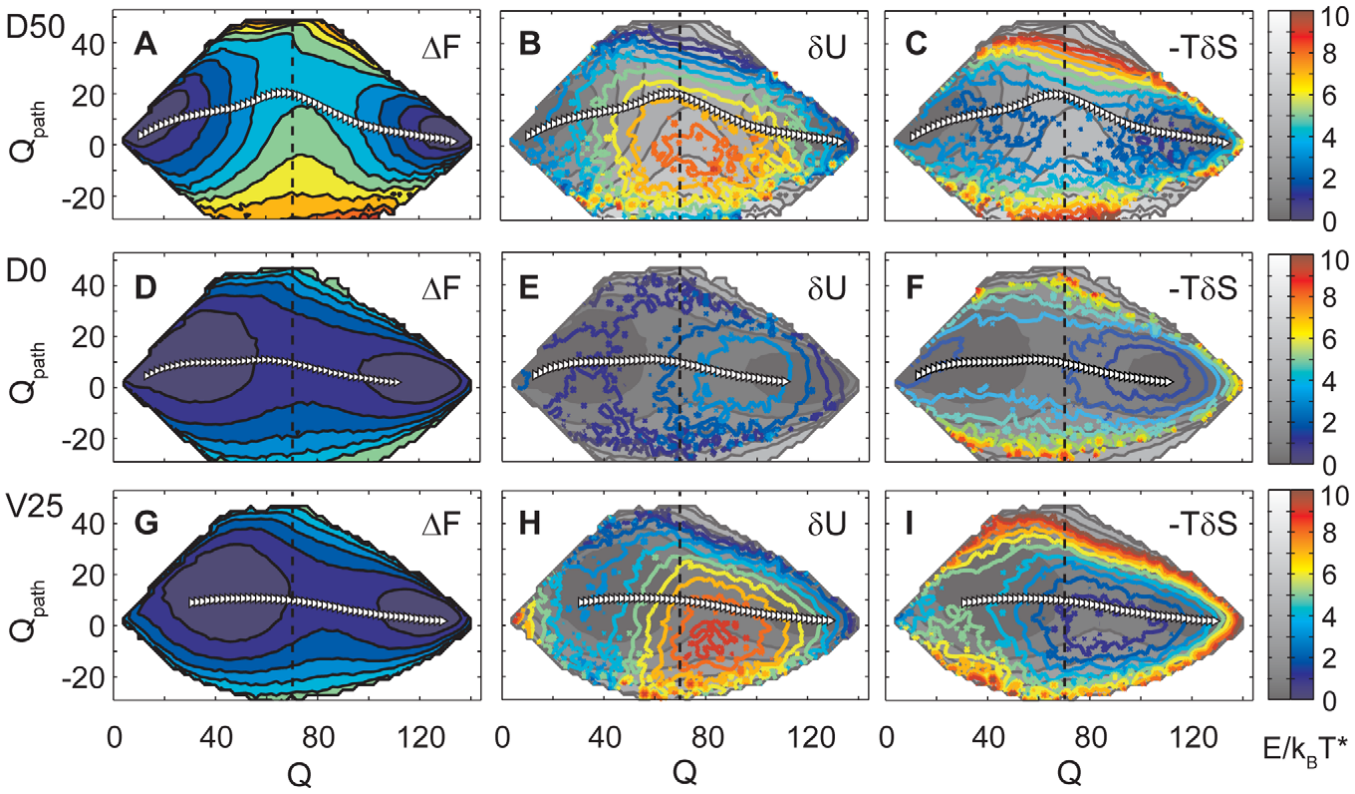
Free energy landscape decompositions 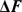, 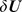 and 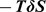 for systems with modified interactions. **A**–**C** D50: 50% softer dihedral potentials. **D**–**F**: D0: no dihedral potential. **G**–**I** V25: bead size 25% of normal. As in Fig. 2, landscapes are shown as a function of the total number of formed contacts 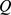 and of the coordinate 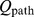 that quantifies the pattern of contact formation. Positive values correspond to the pattern in the WT-dominant transition state, negative values mean a reversed pattern. Average folding paths are marked by white triangles. The system D50 retains the WT-dominant pattern of contact formation in its transition state, indicated by positive values of 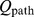. Both energy and entropy contributions to the landscape follow the same qualitative picture as in the full model. Again, the bias in 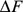 towards WT-dominant transition states with positive 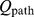 arises from a peak in the energy contribution at negative 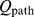, while the entropy contribution provides no such bias. In the system D0 the biasing peak in the energy contribution 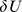 is lost. The entropy again provides no bias either. The resulting free energy landscape has a very low broad barrier and leads to an unspecific mechanism. A very similar free energy landscape with low barrier also results for the system V25, which also folds with an unspecific mechanism. Here the biasing feature in the energy contribution 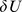 to the landscape is clearly present, but the entropy contribution 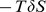 is now modified to counteract it.

To ascertain the role of different interaction terms in shaping the transition state structure we have repeated the analysis of the free energy landscape for models with perturbed interactions. To reduce the extent of excluded volume we have created a system with smaller beads, designated V25, where the chain occupies only 25% of the volume that it has in the original model. To reduce the energy cost for distorted structures we have built systems with weakened dihedral potentials, named D50 and D0, where the dihedral term has 50% of its original strength, or is removed entirely. Free energy landscapes for these perturbed systems together with the residual energy and entropy contributions, analogous to those in Fig. 2, are shown in Fig. 5. The landscape for system D50 is qualitatively similar to that for the original system. Its unfolded and folded basins are connected by a curved folding route with a transition state characterized by positive 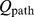. The bias in the transition region arises again from a peak in the energy contribution, while the entropy provides no corresponding feature. But both the free energy barrier and the underlying peak in the energy contribution are smaller than in the original model. In the system D0 without dihedral interactions the energy peak in the off-pathway transition region is lost, and the system folds along a heterogeneous reaction path over a very low barrier of the order of 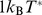 A very similar free energy landscape with a suppressed barrier and a straight folding path also results for the system V25 with smaller chain volume. Unlike in the previous case without dihedral interactions, the peak in 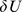 at negative 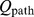 is preserved, with similar strength to the original system. A neutral free energy landscape arises because the entropy landscape now possesses a pronounced corresponding basin that compensates the effect of the energy peak.

**Figure 6:**
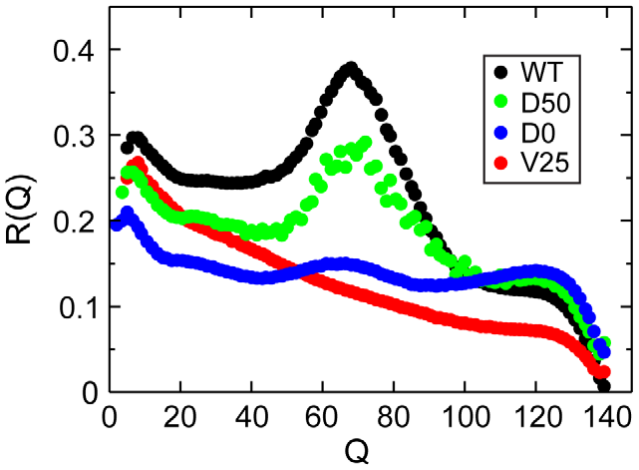
Loss of folding mechanism. The route measure, which quantifies the specificity of the folding pathway, is plotted as a function of folding progress 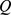 for the full structure-based model and for the systems D50 and D0 with softer dihedral potentials and V25 with reduced bead size. The peak near 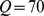 for the WT model reflects the specific transition state. In the system D50 with softer dihedral potentials, the transition state is preserved, although with lowered specificity. In the absence of any dihedral potential in the system D0 only very little specificity remains in the transition state. In the system V25 with reduced bead size, the specificity of the transition state is also completely lost and folding takes place on a variety of routes.

The link between the observed changes in the free energy landscapes for the modified systems and their folding mechanisms is highlighted by the route measure [15], [40] (Fig. 6). It is defined as
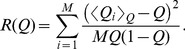
(7)

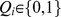
 specifies the formation of contact 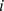 in a particular structure, and the average is taken over all configurations with 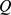 contacts formed out of the total 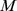. The route measure is the normalized variance among the formation probabilities 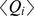 for all contacts computed at a particular value of 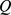. High values of 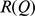 up to unity signal a specific pattern of contact formation, where a subset of contacts are preferentially formed over the rest, while values close to zero correspond to an unspecific mechanism with an equal probability of formation for all contacts. The specific pattern of contact formation realized in the polarized transition state of the original folding model is reflected by a peak in the route measure. This peak is reduced for the sytem D50, where weakened dihedral potentials lead to a lowered barrier and a less pronounced bias towards the WT-dominant mechanism. For the systems D0 and V25, where the loss of dihedral potentials or of chain volume have suppressed the structure of the free energy landscape down to a remnant barrier of the order of only 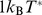, the route measure also indicates unspecific folding with unconstrained mechanisms.

### Structural characterization of competing pathways

We return to the original model in order to study the structural basis for the energy bias in favor of the WT-dominant transition state. From our simulation trajectories with the original model we have identified all structures in the transition region, defined by a total number of formed contacts 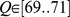, and classified them according to their pattern of contact formation measured by . The trajectories contain 28509 on-pathway structures with 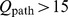, and 537 off-pathway structures with an inverted pattern of contact formation characterized by 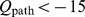. In between these two extreme alternatives we also collected 1672 structures with an unpolarized contact map described by values of 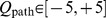.

**Figure 7:**
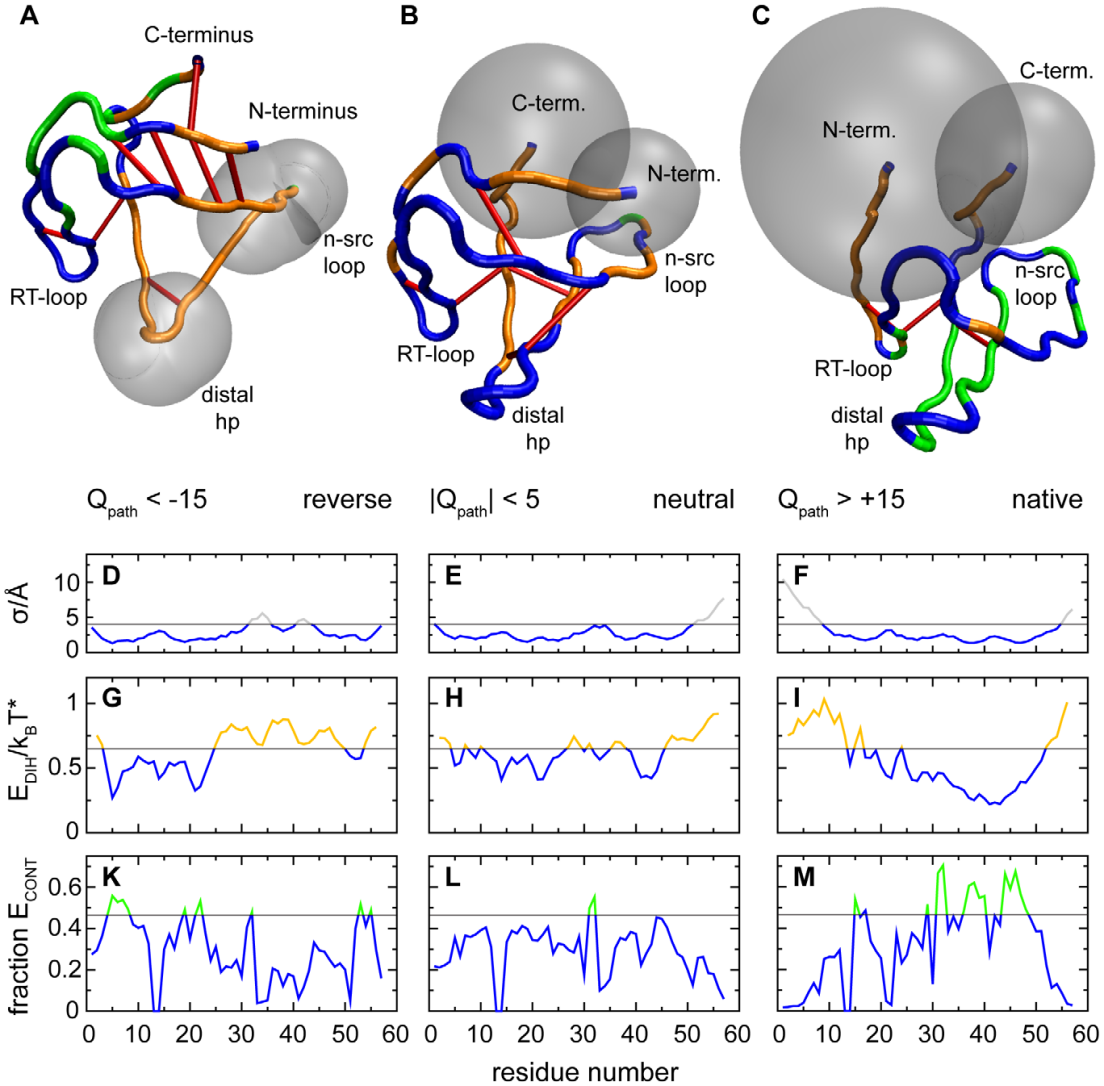
Properties of on- and off-pathway transition states. Transition state configurations obtained at 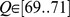 were classified as reversed, neutral or on-pathway according to their mechanistic coordinates of 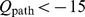, 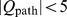 or 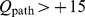, respectively. Averaged structures are shown in panels **A**–**C**. Residues with a high average fraction of formed contacts are colored green, regions with strongly distorted dihedral angles are marked in orange. Translucent spheroids give the standard deviations from the average position for residues whose locations vary strongly between configurations. Strong repulsive interactions are marked in red. The underlying data are plotted in the graphs below each structure; with standard deviations of positions in panels **D**–**F**, dihedral energies in panels **G**–**I**, and the fraction of formed contacts in panels **K**–**M**. Horizontal bars indicate the highlighting thresholds. (Figure prepared with VMD [42].).

The average structures for each group, shown in Fig. 7, reflect the structural preferences of the chosen contact maps. Regions of the chain that are supported by formed contacts adopt consistently native-like local structures, while the rest of the chain remains flexible and takes on various distorted configurations in the individual selected structures. This flexibility is represented in the figure by translucent grey spheres, which indicate the standard deviation of the chain positions in the selected set. The standard deviation of residue positions is also plotted below each average structure, in graphs **D**–**F**, and the regions with high variation represented in the structures are indicated. Plots **K**–**M** give the fraction of formed native contacts for each residue in the three sets of transition state structures. Residues with a high fraction of formed native contacts are marked in green in the shown average structures. In the average on-pathway transition state **C** the central three-stranded beta sheet is consistently well formed, while the termini remain mobile. In the average off-pathway structure **A** the inverted contact map stabilizes the termini and the hairpin formed by the RT-loop, while the central strands retain their flexibility. In the structures with neutral contact maps, shown in panel **B**, the complete chain is in an approximately native-like configuration, with most of the remaining variation at the termini. Red lines in the average structures mark residue pairs with strong repulsive interactions. As non-native interactions, repulsive contacts are only possible between distorted regions of the chain. The strongest repulsive interactions do however not occur in the highly flexible regions of each structure. Instead they are localized in partially structured regions, where parts of the chain are already constrained in mutual proximity. In any case the quantitative contribution of repulsive interactions to the penalty for off-pathway transition states is small.

The average dihedral energies along the chain, plotted in graphs **G**–**I**, which make one of the largest contributions to the bias towards native transition states, closely reflect the pattern of structured and unstructured regions in each set of structures. In the average structures, regions of high dihedral energy are marked in orange. In every case the native-like regions have low average dihedral energies, and the distortions in the unstructured regions lead to high dihedral energies. The neutral contact map results in a relatively even pattern of dihedral energies. The patterns for the states with WT-dominant and inverted contact maps are complementary. In both the on- and the off-pathway states the variance in dihedral energies between native-like and distorted residues exceeds 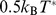. This variation inside each structure masks the smaller systematic difference in the averages on the order of 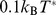 that gives rise to the overall stabilization of WT-dominant structures compared to off-pathway states. Similarly small distortions of formed contacts are responsible for the contribution in favor of WT-dominant transition states that is made by contact energies.

### Cooperative stabilization of the native TSE

**Figure 8:**
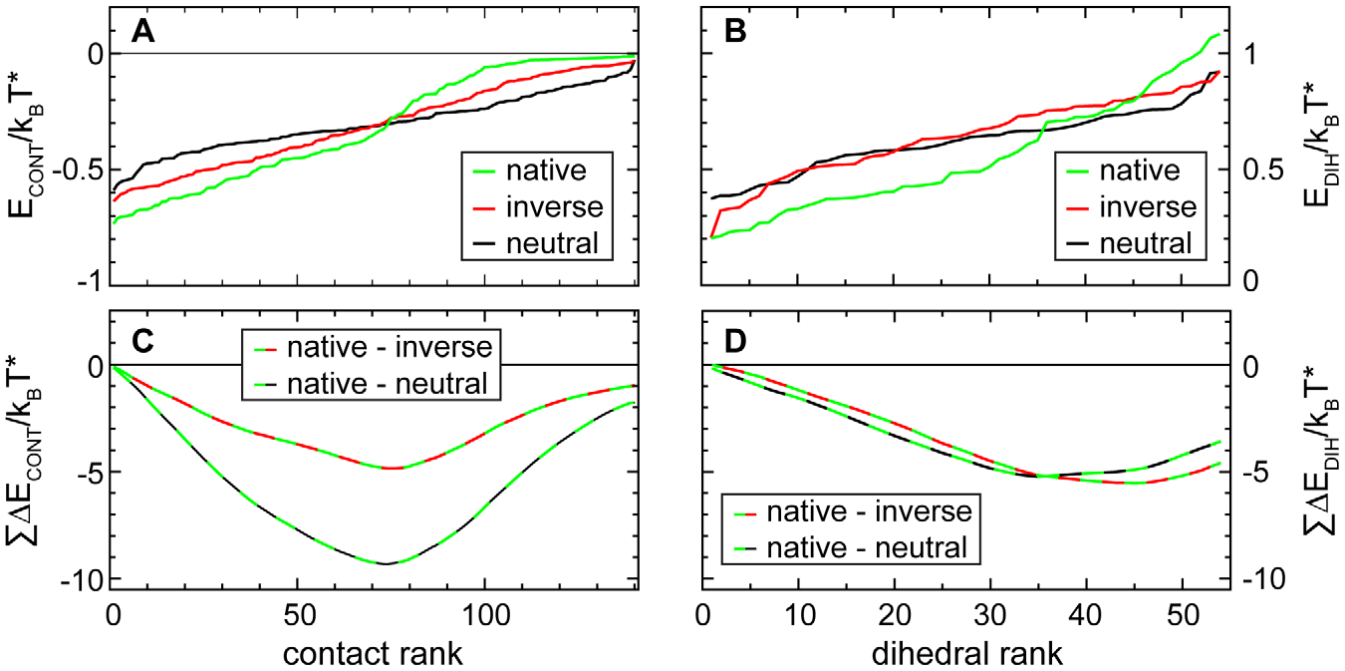
WT-dominant transition states are favored over off-pathway structures by dihedral- and contact energies. Average contact energies and dihedral energies for each residue are shown ordered from lowest to highest in panels **A** and **B**, for on-pathway transition states and for both neutral and reversed off-pathway structures. Both the lowest and the highest residue energies occur in WT-dominant transition state structures. Residues that are stabilized in native transition structures have lower energies than the most stabilized residues in either reversed or neutral off-pathway states, and the most destabilized residues in WT-dominant transition state structures have higher energies than any destabilized residues in any off-pathway structures. The pattern of dihedral energies is very similar in neutral and in reversed off-pathway structures, which both lack the very stable and also the highly unstable residues found in WT-dominant transition states. For contacts, the spread from lowest to highest energies is lowest in neutral structures, larger in reversed states, and largest again for the WT-dominant transition structures. The cumulative differences shown in panels **C** and **D** confirm that in the balance the low energies from the majority of better-stabilized residues in WT-dominant transition states outweigh the higher energies from the few strongly destabilized ones.

In Fig. 8 the average energies contributed by each of the contacts and dihedrals in the three different sets of transition state structures are analyzed directly, without reference to their position or role in the structure. Instead the 140 native contacts and their energies are ranked in Fig. 8 **A** in order of their average stabilization from most to least stabilized. In panel **B** the average dihedral energies are plotted in the same way, ranked from the lowest to the highest average values. In this arrangement it becomes apparent that the stabilization of WT-dominant transition state arises from the most native-like contacts and dihedrals. The most favorable contacts and dihedrals in on-pathway transition states have the absolutely lowest average energies obtained in any of the three groups of configurations. Although the absolutely least favorable contact and dihedral energies also occur in WT-dominant states, the cumulative energy differences plotted in panels **C** and **D** confirm that the WT-dominant transition state structure is in the balance stabilized over the alternatives.

To achieve this stabilization the contacts that are formed in the WT-dominant transition state and the portions of the chain that adopt native configurations must reach lower energies than alternative chain segments or native contacts that are formed in the neutral and inverted off-pathway states.

**Figure 9:**
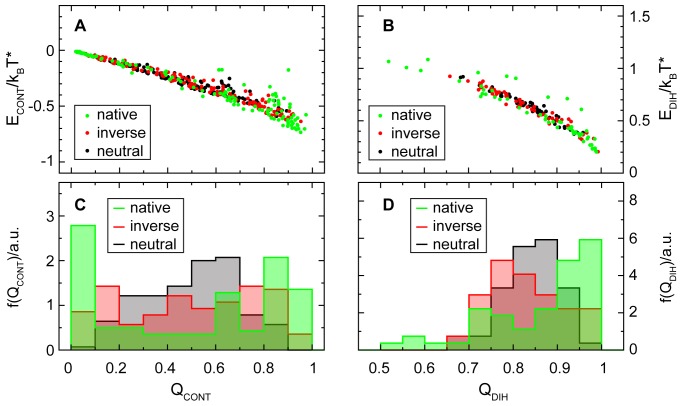
Cooperative stabilization of highly formed structures. The average energy contribution from each contact is plotted against the fraction 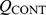 of structures with the contact formed in panel **A**, for on-pathway transition states and for both reversed and neutral off-pathway structures. Similarly the average energy from each dihedral is plotted in panel **B** against the fraction 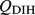 of structures with the dihedral in its native-like rotamer state, again for on-pathway transition states and for reversed and neutral off-pathway structures. Both contact and dihedral energies naturally decrease with the fraction of native-like local structures. Due to remaining distortions the average stabilization is generally smaller than it would be expected from perfectly native configurations. For frequently formed contacts and dihedrals the downward curvatures of the plots however indicate an added gain in stabilization beyond their increase in frequency. While more rarely formed contacts and dihedrals also remain relatively more distorted even in native-like structures, dihedrals and contacts that are frequently native-like also come closer to their native configuration when they are formed. This relationship appears equally in on-pathway transition states and in off-pathway structures. Histograms of 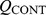 and 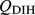 shown in panels **C** and **D** reveal that on-pathway transition states benefit most from this additional stabilization of frequently native-like contacts and dihedrals, thanks to broadened or even bimodal distributions that maximize the fraction of such very frequently formed contacts and dihedrals.

Fig. 9 shows a general trend in the energies of individual contacts and dihedrals that forms the basis for this energetic distinction between on- and off-pathway transition states. Energies of individual contacts and dihedrals are plotted in panels **A** and **B** against the probability that the contact pair or the chain segment is in a native configuration. For contacts this probability to be native is simply the probability that the contact is formed, called 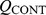. For the dihedral energies the probability that the dihedral has a configuration in the native basin of its torsional potential is used to define the probability to be native, 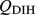. These probabilities and the corresponding energies of the contacts and dihedrals are plotted separately for the groups of transition state structures with on-pathway, neutral and inverted contact maps. Both for contacts and for dihedrals, all three sets of structures show a similar behavior of the energies.

Under the influence of the native-centered stabilizing potentials of the structure-based model, the average energy of each contact and dihedral decreases with increasing probability to be native-like. Against idealized expectations the plots show a significant downward curvature. Contacts and dihedrals that are more frequently native-like receive an additional stabilization that is eventually responsible for the stabilization of WT-dominant transition states. Ideally, a contact would not be stabilized at all when it is unformed, and it would be perfectly native and stabilized by the full contact potential every time it is formed. In this case its average energy would just be the total stabilization of a formed contact multiplied with the fraction of time spent with the contact formed. Imperfect stabilization of a formed contact, caused for example by fluctuations around the native pair distance, would reduce the stabilization. A nonzero degree of stabilization for unformed contacts could equally reduce the energetic advantage of contact formation. But as long as every situation when a contact is formed or unformed remains equivalent to all other instances of contact formation or breaking, the stabilization would still be proportional to the fraction of times when the contact is formed. Instead the observed curvature in the plots proves that some contacts are better formed than others and thus receive additional stabilization. Specifically contacts that are formed frequently are at the same time also more closely native-like, and their stabilization is thus further increased. The same holds for dihedrals, which are distorted on average by a smaller amount when they are also distorted more rarely.

WT-dominant transition state structures are favored by this effect, because they contain a higher proportion of contacts and dihedrals receiving the extra stabilization. The distributions of 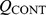 and 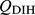, plotted in Fig. 9 **C** and **D**, are broadened or even bimodal in the WT-dominant transition state structures, while they are narrower in the structures with inverted, or especially with neutral, contact maps. For a given total number of formed contacts, a bimodal distribution will tend to maximize the fraction of frequently formed contacts in the structure and thus benefit from their extra stabilization.

## Discussion

Against expectations, the transition state structure for the folding of SH3 is not determined by the configurational entropy of the protein chain, even though the interaction potential is generic and simplified, and chain connectivity is a prominent feature of the model. The known agreement between the folding routes of different SH3 domains confirms that the mechanism is determined by the geometry of the fold. But the geometry does not select the WT-dominant transition state based on its entropy. Instead, the competing chain geometries in the transition region are discriminated by their energies.

The stabilizing effect that was found in contact and dihedral potentials favors structured transition states over random contact formation in general. Any pattern of structure formation that prefers one set of contacts over the others will lead to a broadened or even bimodal distribution of formation probabilities, which will benefit from the extra stabilization of frequently-formed native structures.

The underlying cause of this effect is probably cooperativity. In both the WT-dominant and the inverted transition state structures shown in Fig. 7, native-like contacts and dihedral conformations are concentrated in contiguous parts of the native structure. It seems plausible that native contacts and dihedrals receive an extra stabilization in this situation, because adjacent contacts further restrict deviations from the native configuration, especially if multiple contacts act on the same residue.

Cooperative stabilization of native-like structure is an essential ingredient of protein folding in general [41]. Cooperativity is required to predict realistic barrier heights in folding simulations, and it is necessary to obtain a stable native state in structure-based models. Cooperativity is implicitly present in the model because the adoption of native-like backbone configurations, guided by the local dihedral potential, automatically leads to the simultaneous formation of native contacts. The mutual stabilization of native-like structure in adjacent parts of the protein is an additional consequence of this built-in cooperativity between the different elements of the potential.

Such concurrent stabilization of native structure by several potential contributions is a fundamental characteristic of the protein energy landscape, it is a consequence of the principle of minimal frustration. A tendency to fold with polarized transition states, like the one of SH3, is therefore deeply rooted in proteins in general.

In our model, the WT-dominant transition state and the off-pathway structure with the inverse polarization are both favored by the observed stabilization of contiguous native structure. The WT-dominant transition state is favored over the alternative only quantitatively. According to Fig. 8 the decisive energetic advantage for the WT-dominant transition state is provided by the dihedral potential. Inspection of the average conformations in Fig. 7 suggests a structural reason. In the WT-dominant transition state the native structure is formed by the central part of the chain, and the termini remain unstructured. In the inverse case the termini are joined by their native contacts, and the central segment of the chain is unstructured. But this central segment of the chain is nonetheless strongly constrained by its connections to the bound termini, while the free termini in the WT-dominant transition state are naturally separated by the interposed native core. In the WT-dominant transition state the termini can thus be unbound and still maintain relatively native-like conformations individually. In the off-pathway state, only strongly distorted configurations of the central chain segment can avoid the formation of additional native contacts in this region. The distortion affects primarily the dihedral potentials and the angles, but it will also propagate to the formed contacts. The forced distortion of the central chain segment in the off-pathway transition state is then responsible for the increased energies observed in both the backbone potentials and the contact interactions in off-pathway structures.

In the context of the larger kinase the SH3 domain may still be forced to fold with the penalized inverted mechanism, depending on when SH3 folds in relation to the neighboring domain. If the SH3 domain folded after the rest of the protein, the termini would be held close together by the existing structure, and the folding barrier would be increased. Once the protein is completely folded, the higher barrier for unfolding with joint termini could on the other hand increase the kinetic stability of the SH3 domain.

The energy cost for distortions of the backbone opposes the effect of configurational entropy. In an ideal protein, as it is recreated by a structure-based model, the native structure is the global energy minimum. Accessible non-native configurations, which increase the configurational entropy of the system, are only reached at the cost of increased potential energy. Globally the compensation of energy and entropy is a central feature of the folding reaction. In the competition between alternative folding routes, the higher energy of additional distorted configurations will counter their favorable contribution to the configurational entropy. For SH3 the energy cost of distortion outweighs the entropic effects, and the folding route is selected by the energy terms in the model.

Quantitatively the relevant energies are not very strong. Contributions of the order of 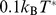 for individual contacts and dihedrals add up to an apparent free energy bias in favor of the WT-dominant route of 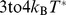 seen in Fig. 2 **A**. A reduction of the underlying dihedral interactions by 50% is sufficient to reduce this bias to 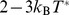 in Fig. 5 **A**. The effect of this loss on the mechanism is clearly visble in the route measure in Fig. 6, and in the absence of dihedral interactions the remaining contribution from the contact potential is not sufficient to maintain the mechanism. Individually the isolated differences in the contact and dihedral energies observed between the extremes of positive and negative 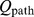 reach 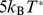 each. The smaller apparent free energy bias already contains a competing entropy contribution, visible in Fig. 3, that also reaches several 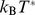. Through manipulation of the chain volume this entropy contribution too can be modified sufficiently to change the folding route. A new basin in the entropy contribution to the landscape of 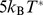 compensates enough of the persisting energy bias to suppress the folding mechanism. Similar changes in energy are also readily accessible in actual proteins. Individual contacts provide energies of the order of 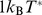, and a small number of mutations could easily achieve a sufficient redistribution of the stabilization to modify the mechanism. The partial cancellation of the configurational entropy by the energy cost of distortions facilitates such adjustments by reducing the required energies.

In conclusion, the observed folding mechanism of SH3 in our simulations is not explained by the configurational entropy of the chain. Instead a polarized folding mechanism is favored by cooperative interactions that stabilize contiguous native structure. Chain connectivity is responsible for the mechanism because it provides local coupling, not because it determines configurational entropy. The actual structure of the WT-dominant transition state ensemble is determined by the energy cost of distorted backbone configurations, which acts directly against the trend given by chain entropy. Although the folding mechanism is determined by an unexpected energetic effect, the results illustrate again the malleability of the folding mechanisms and functional dynamics of proteins. Small, accessible changes to several competing energy and entropy contributions can each reverse the weak bias towards the observed mechanism and may serve to switch between alternative behaviors.

## Supporting Information

Figure S1
**Surfaces** 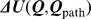 **and** 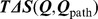 **.** The global decrease along 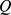 dominates both contributions. It obscures finer structures that determine the features of 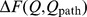, which control the folding mechanism. (Data shown for the unperturbed model).(PDF)

Figure S2
**Fit to describe the** 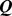 **-dependence of** 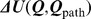 **and** 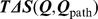 **.** The shapes of both surfaces are averaged along 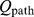 to obtain the plotted one-dimensional functions 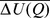 and 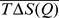. To describe the common decrease of both terms, a quadratic function 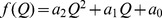 is fitted to both 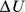 and 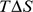 together. This is equivalent to fitting to the average of both terms, 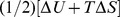, which is also plotted. (Data shown for the unperturbed model).(PDF)

Text S1
**Determination of the overall bias along** 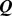 **in** 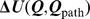 **and** 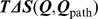 **.** Additional technical information about the fitting procedure used to determine 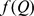, defined in Eqn. 6.(PDF)
