# Supplementary figures and images for: The Dominant Folding Route Minimizes Backbone Distortion in SH3
Source: PLoS Comput Biol. 2012 Nov 15;8(11):e1002776. doi: 10.1371/journal.pcbi.1002776 (PMC3499259; doi:10.1371/journal.pcbi.1002776)

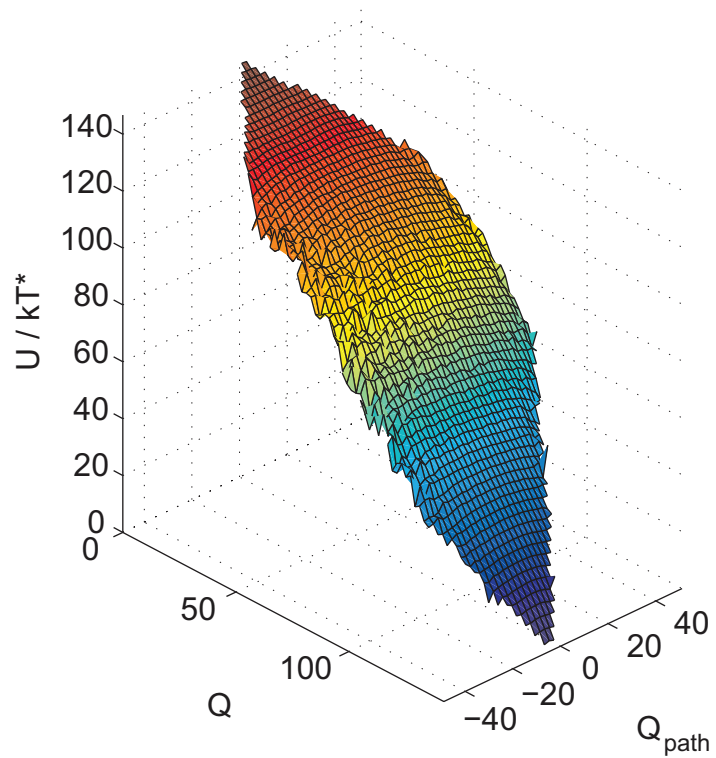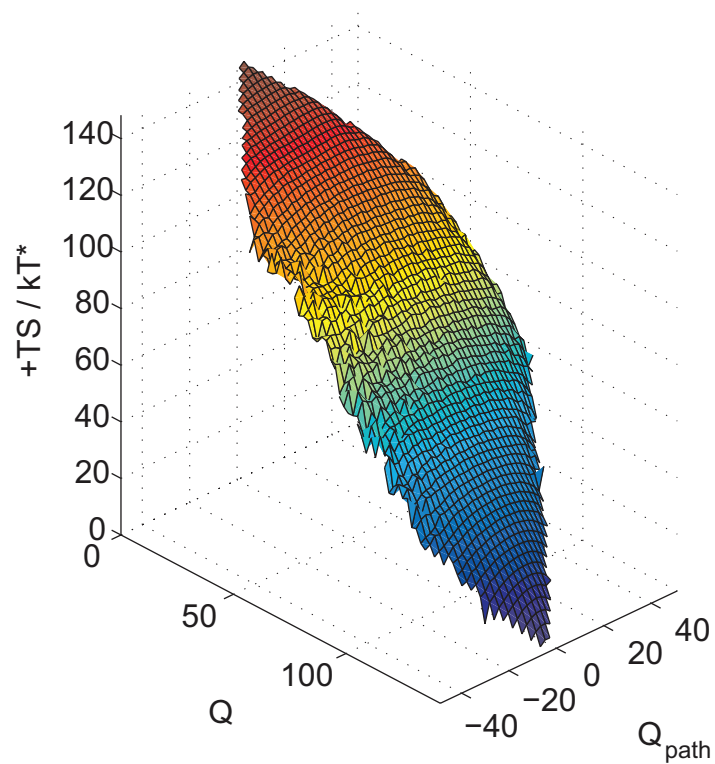

Supplement: Figure S1 — Surfaces and . The global decrease along dominates both contributions. It obscures finer structures that determine the features of , which control the folding mechanism. (Data shown for the unperturbed model). (PDF) [file pcbi.1002776.s001.pdf]

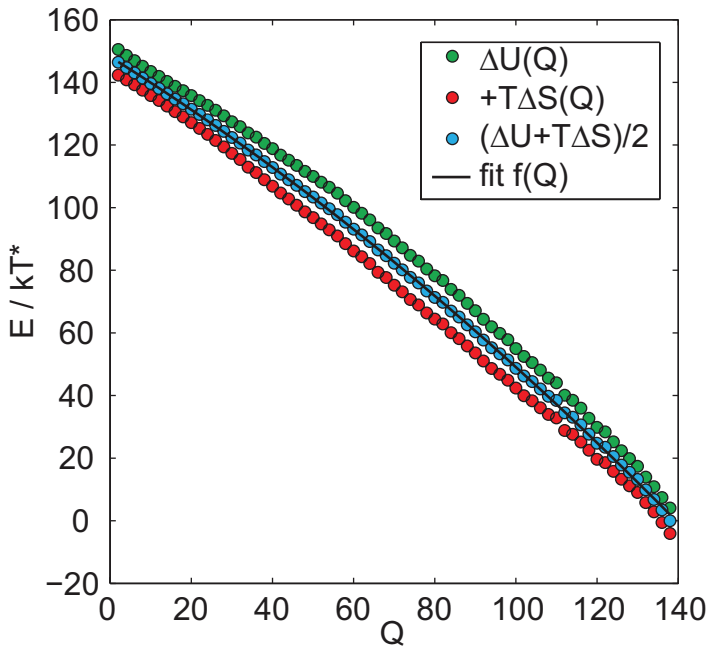

Supplement: Figure S2 — Fit to describe the -dependence of and . The shapes of both surfaces are averaged along to obtain the plotted one-dimensional functions and . To describe the common decrease of both terms, a quadratic function is fitted to both and together. This is equivalent to fitting to the average of both terms, , which is also plotted. (Data shown for the unperturbed model). (PDF) [file pcbi.1002776.s002.pdf]
