# Supplementary material for: The Dominant Folding Route Minimizes Backbone Distortion in SH3
Source: PLoS Comput Biol. 2012 Nov 15;8(11):e1002776. doi: 10.1371/journal.pcbi.1002776 (PMC3499259; doi:10.1371/journal.pcbi.1002776)
Supplement: Text S1 — Determination of the overall bias along in and . Additional technical information about the fitting procedure used to determine , defined in Eqn. 6. (PDF) [file pcbi.1002776.s003.pdf]

Supplementary Text S1 for the article:
The Dominant Folding Route Minimizes Backbone Distortion in SH3
by H. Lammert, J. K. Noel and J. N. Onuchic

We study the free energy landscape $\Delta F(Q, Q_{\mathrm{path}})$ determined from our simulations and the underlying energy and entropy contributions $\Delta U(Q, Q_{\mathrm{path}})$ and $T\Delta S(Q, Q_{\mathrm{path}})$. The full contributions $\Delta U(Q, Q_{\mathrm{path}})$ and $+T\Delta S(Q, Q_{\mathrm{path}})$ are plotted in Fig. S1. Both plots are dominated by the gradient along the reaction coordinate Q, which describes the overall progress of the folding reaction. Any structure in the direction of Q_{path}, which describes the folding mechanism, is obscured. In order to reveal the superimposed structure that is shaping the folding mechanism we remove the global gradients along Q. In Eqn. 6 we define a function $f(Q)$ that contains the common portion of ΔU and $T\Delta S$ that is canceled in their subtraction to form ΔF. The complete information about the mechanism is contained in the residuals $\delta U(Q, Q_{\mathrm{path}}) = \Delta U(Q, Q_{\mathrm{path}}) - f(Q)$ and $T\delta S(Q, Q_{\mathrm{path}}) = T\Delta S(Q, Q_{\mathrm{path}}) - f(Q)$. $f(Q)$ is obtained from a fit to ΔU and $T\Delta S$.

Although the dominant contribution to the potential is contact energy, and Q is simply the number of formed contacts, the observed Q-dependence of the energy is not linear. The energy and entropy terms possess enough curvature to necessitate the use of a quadratic function $f(Q) = a_2Q^2 + a_1Q + a_0$.

In order to obtain a fair estimate of the common component of energy and entropy we fit $f(Q)$ to both ΔU and $T\Delta S$ together. For a least-squares fit this is equivalent to fitting to the average of both terms, $(1/2)[\Delta U + T\Delta S]$.

All points on both the surfaces $\Delta U(Q, Q_{\mathrm{path}})$ and $T\Delta S(Q, Q_{\mathrm{path}})$ enter equally into the fit. This is different from a fit to the one-dimensional functions $\Delta U_{\mathrm{1D}}(Q)$ and $T\Delta S_{\mathrm{1D}}(Q)$ that could be obtained as components of the one-dimensional free energy profile $\Delta F_{\mathrm{1D}}(Q)$, where points at different Q_{path} enter with different thermodynamic weights. Instead it corresponds to a fit to the direct averages along Q_{path}, which give a beter description of the overal shape of the two-dimensional surfaces, i.e. $\overline{\Delta U(Q)} = (1/N)\sum_{i=1}^{N} \Delta U(Q, Q_{\mathrm{path},i})$ for the energy.

The one-dimensional functions $\overline{\Delta U(Q)}$ and $\overline{T\Delta S(Q)}$ for the original, unperturbed model are shown in Fig. S2 together with their average and with the fitted quadratic function $f(Q)$.
